# Mitophagy for cardioprotection

**DOI:** 10.1007/s00395-023-01009-x

**Published:** 2023-10-05

**Authors:** Allen Sam Titus, Eun-Ah Sung, Daniela Zablocki, Junichi Sadoshima

**Affiliations:** Department of Cell Biology and Molecular Medicine, Cardiovascular Research Institute, Rutgers New Jersey Medical School, 185 South Orange Ave, MSB G-609, Newark, NJ 07103 USA

**Keywords:** Mitophagy, Mitochondrial quality control, Alternative mitophagy, Drp1

## Abstract

Mitochondrial function is maintained by several strictly coordinated mechanisms, collectively termed mitochondrial quality control mechanisms, including fusion and fission, degradation, and biogenesis. As the primary source of energy in cardiomyocytes, mitochondria are the central organelle for maintaining cardiac function. Since adult cardiomyocytes in humans rarely divide, the number of dysfunctional mitochondria cannot easily be diluted through cell division. Thus, efficient degradation of dysfunctional mitochondria is crucial to maintaining cellular function. Mitophagy, a mitochondria specific form of autophagy, is a major mechanism by which damaged or unnecessary mitochondria are targeted and eliminated. Mitophagy is active in cardiomyocytes at baseline and in response to stress, and plays an essential role in maintaining the quality of mitochondria in cardiomyocytes. Mitophagy is mediated through multiple mechanisms in the heart, and each of these mechanisms can partially compensate for the loss of another mechanism. However, insufficient levels of mitophagy eventually lead to mitochondrial dysfunction and the development of heart failure. In this review, we discuss the molecular mechanisms of mitophagy in the heart and the role of mitophagy in cardiac pathophysiology, with the focus on recent findings in the field.

## Introduction

Mitochondria are not only the powerhouse of the cell but also a hub for signaling activities that determine the cell’s fate and functionality. Thus, the quality and quantity of mitochondria need to be regulated precisely. Various stress conditions that lead to mitochondrial damage result in leakage of mitochondrial proteins and cause cell death [38, 162]. Repairing damaged mitochondria and eliminating dysfunctional mitochondria are critical to maintaining homeostasis and preventing cell death. Mitophagy is a process by which damaged or unnecessary mitochondria are specifically removed through autophagy-mediated lysosomal degradation, and it is the most well-studied type of selective autophagy [6, 135]. Impaired mitophagy and accumulation of damaged mitochondria lead to cell and tissue damage and are associated with a broad spectrum of pathologies, including neurodegeneration, myopathies, metabolic disorders, inflammation, autoimmune disorders, and cancer [135, 205]. Mitophagy is crucial for maintaining cardiovascular homeostasis and protecting the myocardium. Defects in mitophagy are observed in cardiovascular diseases such as myocardial infarction, cardiac hypertrophy, heart failure, ischemia/reperfusion, and diabetic cardiomyopathy (DCM) [10, 112]. In this review, we focus on the molecular mechanisms that orchestrate selective removal of mitochondria and discuss the role of mitochondrial fission and fusion in mitochondrial quality control mechanisms during myocardial ischemia/reperfusion injury, hypertrophy, and diabetic cardiomyopathy.

### Molecular mechanism of mitophagy

The general steps mediating the selective degradation of mitochondria through mitophagy include the identification and tagging of damaged mitochondria, compartmentalization of the mitochondria by autophagosomes, fusion of the autophagosomes with lysosomes, and proteolysis of the mitochondrial components in the fused autolysosomes [135].

### Mechanism of autophagosome biogenesis

One of the most well-characterized conditions promoting mitophagy is energy stress, where an energy deficiency is sensed and signaled by mammalian target of rapamycin complex 1 (mTORC1) and AMP-activated protein kinase (AMPK) [75]. AMPK and mTORC1 post-translationally modify the unc-51-like kinase 1 (Ulk1) complex (which includes Ulk1, Atg13, Atg101, and FIP200) to initiate formation of the phagophore, a double-membrane structure that sequesters the cargo to be degraded. Class III phosphatidylinositol 3-kinase (PI3KC3) complex I (PI3KC3-C1), consisting of Atg6/Beclin 1, Atg14, PI3KC3/Vps34, and the regulatory subunit Vps15/p150, generates phosphatidyl inositol 3-phosphate (PI3P) to nucleate the phagophore [137]. Autophagosome expansion and nucleation are facilitated by vesicles containing Atg9, the sole multi-membrane-spanning protein in the autophagosome-forming machinery [128]. While autophagosomes are being established, Atg18 and WIPIs (WD-repeat proteins interacting with phosphoinositides), members of the PROPPIN (*β*-propellors that bind polyphosphoinositides) family of proteins, can directly interact with PI3P via a conserved FRRG motif and facilitate the recruitment of downstream Atg effector proteins Atg12-5-16/Atg12-5-16L1 [128]. Atg8/LC3 is a well-known marker protein for traditional autophagy and a ubiquitin-like (Ubl) protein. Atg4 protease immediately processes newly translated LC3 (pro-LC3) proteins at the C-terminus to form LC3-I [176]. LC3 is then activated by Atg7 (E1 enzyme) and transferred to Atg3 (E2 enzyme), and the Atg12-5-16L1 complex (E3 enzyme) facilitates the transfer of LC3 from Atg3 to phosphatidylethanolamine (PE) (Atg8/LC3 lipidation) [130]. Membrane recruitment of the Atg12-5-16L1 complex and Atg8/LC3 lipidation are controlled by the PI3P-binding protein WIPI2B under starvation conditions [128]. The double-membrane autophagosomes, decorated by Atg8 family proteins LC3/GABARAP (GABA type A receptor-associated protein), form isolation compartments for sequestration of various cargos. The specific endosomal sorting complexes required for transport (ESCRT) proteins are recruited to autophagosomes by Atg8s to keep the membrane impermeable and sealed, allowing the autophagosomes to mature into degradative autophagic compartments [68]. In HEK293 and HeLa cells lacking Atg8s, autophagic organelles are permeable, arrest as amphisomes, and do not progress to functional autolysosomes [68]. The Atg8 family proteins, especially GABARAP, promote Pleckstrin homology domain-containing protein family member 1 (PLEKHM1) recruitment and govern autophagosome–lysosome fusion [103, 124]. Phagophore expansion occurs through the action of Atg9 [102]. Rab7 small GTPase directs the trafficking of autophagosomes along microtubules and facilitates fusion with lysosomes to degrade the cargo materials via acidic hydrolases [63]. The fusion of mature autophagosomes to lysosomes is also facilitated by SNARE proteins [177]. The lysosome-associated membrane proteins (LAMP-1 and LAMP-2) promote acidification of lysosomes by directly interacting with and inhibiting the lysosomal cation channel TMEM175, thereby maintaining the acidic pH required for hydrolase activity [221]. Conventional forms of mitophagy utilize a similar mechanism of autophagosome formation as that used for non-selective autophagy, as described above. However, the mechanisms of phagosome initiation and progression differ in unconventional forms of mitophagy and are discussed in Sect. “Alternative mitophagy and mechanism”.

### Mode of selection of mitochondria

**Fig. 1:**
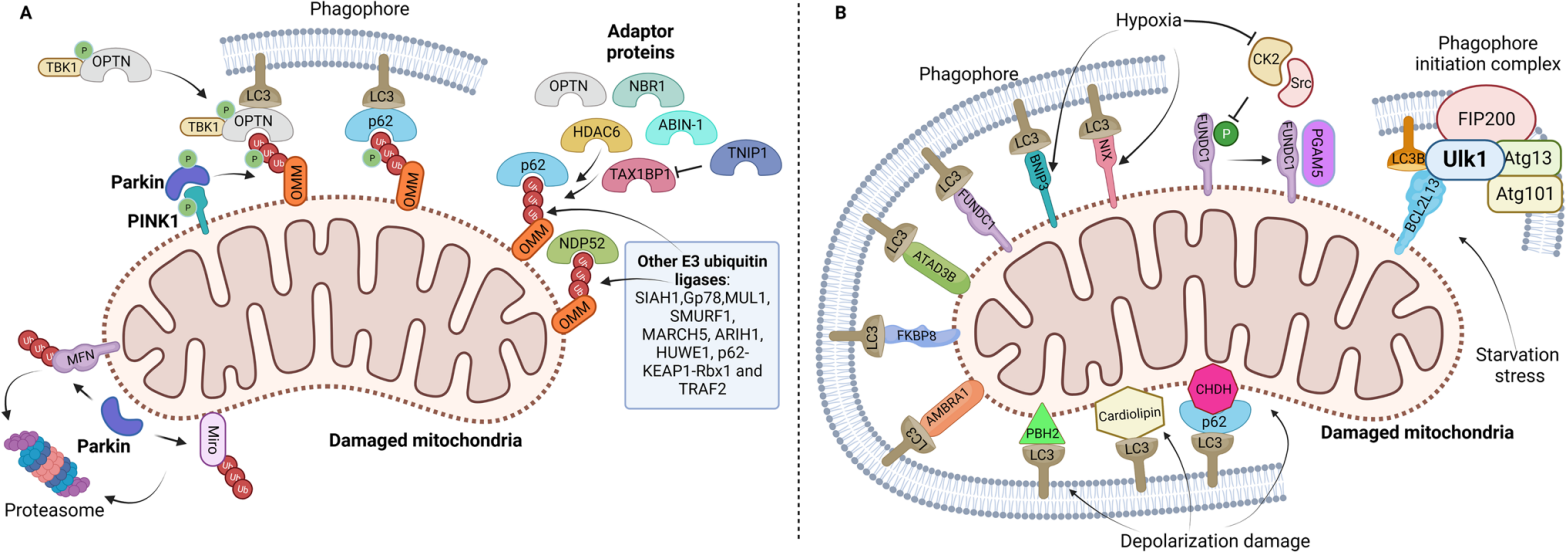
Mechanisms of mitophagy. **A** Ubiquitin-dependent mitophagy. In the PINK1–Parkin pathway of mitophagy, PINK1 is stabilized on the OMM following stress, which then stimulates Parkin recruitment. Several components of the outer membrane are ubiquitinated by Parkin. Following this, PINK1 phosphorylates poly-Ub chains, acting as an "eat me" signal for the autophagic machinery. Phosphorylated poly-Ub chains on mitochondrial proteins are recognized by adaptor proteins (p62, OPTN, TAX1BP1, NBR1, NDP52, HDAC6, and ABIN-1 or TNIP1), which then bind to LC3 to start the formation of autophagosomes. TNIP1 has been shown to inhibit TAX1BP1 by competition and downregulate mitophagy. By phosphorylating OPTN, TBK1 increases the protein's ability to bind to Ub chains. A feed-forward mechanism promoting mitochondrial clearance is established by the OPTN-TBK1 complex. Prior to mitophagy, alternative E3 ubiquitin ligases that target OMM proteins include Gp78, SMURF1, MUL1, SIAH1, ARIH1, MARCH5, HUWE1, p62-Keap1-Rbx1, and TRAF2. **B** Receptor-mediated mitophagy. Mitophagy receptors BNIP3, NIX, FKBP8, BCL2L13, AMBRA1, ATAD3, and FUNDC1 localize to the OMM and interact with LC3 directly to mediate mitochondrial elimination. BCL2L13 has also been shown to activate mitophagy by recruiting the Ulk1/FIP200/Atg13/Atg101 initiation complex and LC3B during starvation stress. Following mitochondrial depolarization, PHB2 and cardiolipin are externalized to the OMM and interact with LC3. Choline dehydrogenase (CHDH) accumulates in the OMM of depolarized mitochondria and interacts with p62 and LC3. FUNDC1 phosphorylation status is influenced by PGAM5 phosphatase, CK2 and Src kinases, all of which control mitochondrial dynamics during hypoxia. The figure was created with Biorender.com

The selection of mitochondria for degradation via mitophagy can be classified as (a) ubiquitin dependent or (b) ubiquitin independent or receptor mediated [73] (Fig. 1).

#### Ubiquitin-dependent mitophagy

i. PINK1–Parkin-mediated mitophagy
  One of the most well-studied mechanisms of targeting mitochondria for mitophagy occurs through the mitochondrial membrane kinase PTEN-induced putative kinase-1 (PINK1) and the cytosolic E3 ubiquitin ligase Parkin [139]. In functional mitochondria, PINK1 is transported to the inner mitochondrial membrane (IMM) and cleaved by several proteases [156]. During mitochondrial damage and mitochondrial membrane potential dissipation, the translocation to the IMM does not take place and PINK1 is stabilized on the outer mitochondrial membrane (OMM) [156]. This leads to autophosphorylation and activation of PINK1. PINK1 phosphorylates both polyubiquitin at Ser65 and Parkin at Ser65 in the Ubl domain, which in turn induces mitochondrial recruitment and activation of Parkin E3 ubiquitin ligase activity in a feed-forward loop that polyubiquitinates OMM proteins [55, 133].
  Recent studies have shed further light on the molecular mechanism regulating PINK1. Adenine nucleotide translocase 1 and 2 (ANT1 and 2) present on the IMM can induce mitophagy independently of their nucleotide exchange activity by forming a complex with Tim23 and Tim44, leading to stabilization of PINK1 on the OMM [61]. Mitochondrial GSNOR (S-nitrosoglutathione reductase) denitrosylates ANT1 at Cys160, and GSNOR downregulation in cardiomyocytes aggravates mitochondrial dysfunction in the presence of hypertrophic stimuli, accompanied by downregulation of mitophagy [175]. However, how S-nitrosylation of ANT1 at Cys160 negatively regulates mitophagy remains to be clarified. Signaling by RhoA, a small GTPase, promotes stabilization of PINK1 independently of mitochondrial depolarization in cardiomyocytes, thereby promoting mitophagy and protecting the heart against ischemia [163]. In addition, AMPKα2 phosphorylates PINK1 at Ser495 and promotes mitophagy in cardiomyocytes [190].
  Parkin ubiquitinates several OMM proteins, including mitofusin1 (MFN1), mitofusin2 (MFN2), MIRO, and voltage-dependent anion channel (VDAC) [45, 46, 141, 174, 192]. Unintentional ubiquitination and targeting for mitophagy is controlled by deubiquitinating enzymes, including USP8, USP15, USP30, and USP35 [24, 35, 47, 193]. However, phosphorylated ubiquitin and polyubiquitin chains are not recognized by deubiquitinases, thus stabilizing the mitophagy signal [55]. It is of great interest to clarify how endogenous deubiquitinating enzymes contribute to the regulation of mitophagy in the heart. PINK1-dependent MFN2 phosphorylation also promotes the recruitment of Parkin to mitochondria and mediates elimination of fetal cardiomyocyte mitochondria through mitophagy during perinatal development. This allows fetal mitochondria to be replaced with adult ones so that metabolic maturation of individual mitochondria is achieved [50].
  Taken together, accumulating evidence points to the importance of PINK1 and Parkin in targeting mitochondria for degradation in response to various developmental cues and under stress conditions. However, since some of the studies described above used carbonyl cyanide m-chlorophenyl hydrazone (CCCP), a chemical inducer of mitochondrial depolarization and integrated stress response [77], the degree to which PINK1–Parkin is involved under more physiological stress conditions in various tissues is still largely unknown. It should be noted that Parkin-dependent mechanisms are also utilized in other forms of mitochondrial degradation, including those mediated through Rab5-positive early endosomes [53] and mitochondrial-derived vesicles (MDVs) [104].
  Although PINK1 plays a crucial role in Parkin-mediated mitophagy, increasing evidence suggests that Parkin translocation to mitochondria can occur through other molecular mechanisms as well. Heat shock 70 kDa protein 1L (HSPA1L) enhances Parkin recruitment, while BAG ﻿Cochaperone 4 (BAG4) inhibits Parkin translocation to mitochondria [57]. Heat shock protein 72 (HSP72) translocates to damaged mitochondria and facilitates Parkin recruitment in skeletal muscles [33]. B cell leukemia/lymphoma-2 (Bcl-2)-interacting death suppressor (BIS), also known as Bcl-2-associated athanogene 3 (BAG3), a co-chaperone of HSP70, co-migrates with Parkin to mitochondria and executes mitophagy in cardiomyocytes [171]. On the other hand, the anti-apoptotic Bcl-2 family proteins, Bcl-xL and Mcl-1 (in HeLa cells), and the tumor suppressor protein, p53 (in liver and heart), prevent Parkin translocation to mitochondria [59, 60, 62, 213]. The significance of these mechanisms relative to the PINK1-dependent mechanisms remains to be clarified.
ii. Parkin-independent ubiquitin-mediated mitophagy
  Mice genetically deficient in PINK1 and Parkin exhibit normal mitophagy and cardiac function at young ages, suggesting that PINK1–Parkin-independent forms of mitophagy responsible for the basal turnover of mitochondria may exist in cardiomyocytes [79, 81]. There are several E3 ubiquitin ligases that participate in ubiquitin-dependent mitophagy independently of Parkin. These include glycoprotein 78 (Gp78) [41], SMAD-ubiquitination regulatory factor1 (SMURF1) [27, 134], seven in absentia homolog (SIAH)-1 [169], ARIH1/HHARI [187], MARCH5 [18], HUWE1 [30], p62-keap1-Rbx1 axis [206] and MAPL/MULAN/GIDE/MUL1 [4, 91, 215]. Likewise, TNF-receptor-associated factor 2 (TRAF2) is an E3 ubiquitin ligase that is localized to mitochondria at baseline and during pathological conditions, inducing mitophagy in the heart [98]. TRAF2 is unique in that, besides its involvement in innate immunity through TNF receptor signaling, it activates mitophagy even at baseline and protects the heart against sterile infection by facilitating safe disposal of mitochondrial DNA [98, 150]. These ligases can act independently of Parkin and may be more prominent when the PINK1–Parkin pathway is inhibited [132].
iii. Autophagy adaptors
  The ubiquitin chain on OMM proteins triggers the recruitment of a variety of autophagy adaptors, including TAX1 binding protein 1 (TAX1BP1), Nuclear dot protein 52 (NDP52, also known as CALCOCO2), a neighbor of BRCA1 gene 1 (NBR1), Sequestosome-1 (SQSTM1/p62) and optineurin (OPTN) [132]. These adaptor proteins contain a ubiquitin-binding domain, which enables them to recognize and sequester ubiquitinated cargoes, and an LC3-interacting region (LIR) that allows the recruitment of LC3-coated phagophore membrane around the cargo. Histone deacetylase 6 (HDAC6), another ubiquitin-binding protein, is also involved in mitophagy. However, HDAC6 mediates autophagosome–lysosome fusion rather than cargo recruitment [86, 87]. Parkin-induced mitophagy was unaffected by the absence of p62 in transformed mouse embryonic fibroblasts and HeLa cells, suggesting that p62 is not essential, and other adaptors, including NBR1, appear to have redundant roles in the sequestration of damaged mitochondria [123]. In HeLa cells lacking Parkin, PINK1 can promote the recruitment of OPTN, NDP52, and TAXBP1 to induce mitophagy [85]. OPTN is phosphorylated by TANK-binding kinase 1 (TBK1), allowing increased ubiquitin chain binding and mitophagy in HeLa cells treated with CCCP [111]. NDP52 functions as a redox sensor through its redox-sensitive cysteine residues, which promote disulfide bond formation in HeLa cells under H_2_O_2_-treated conditions. Oligomerization of NDP52 in damaged mitochondria through these disulfide bonds facilitates the recruitment of the autophagy machinery for mitophagy in response to oxidative stress [72].
  Additional LIR-containing proteins, serving as positive or negative regulators of mitophagy, have been reported recently. A20 binding inhibitor of nuclear factor kappa B (NF-κB)-1 (ABIN-1), a polyubiquitin-binding protein, is a positive regulator of mitophagy in HEK293 and HeLa cell lines [107]. In contrast, TNIP3-interacting protein 1 (TNIP1) is an LIR-containing protein that binds to the LC3/GABARAP family of proteins and allosterically to TAX1BP1, a mitophagy receptor, thereby acting as a negative regulator of mitophagy. TNIP1 binding to TAXBP1 prevents the binding of ubiquitinated cargos to TAXBP1. Interestingly, ABIN-1 and TNIP1 are aliases for the same protein; thus, its function appears to be context dependent. It should be noted that TNIP1 can be phosphorylated by TBK1, leading to enhancement of the interaction of TNIP1 with FIP200, a protein in the Ulk1 complex. This interaction then competes with the interaction between FIP200 and TAXBP1, releasing FIP200 from the autophagosome. Thus, phosphorylated TNIP1 can act positively in mitophagy by facilitating the recycling of FIP200 [51]. TNIP1 is one of the few proteins identified as an endogenous negative regulator of mitophagy but its effect appears complex. Further investigation is required to clarify how endogenous TNIP1 function is regulated during stress conditions.
  Mitophagy adaptors also have other important functions relevant for mitophagy. For example, p62 has the ability to phase separate ubiquitinated proteins into a larger condensate. How cargo condensation and mitophagy is linked is poorly understood, but it is known that ubiquitinated Nur77 forms condensates that can sequester damaged mitochondria and targets cargo mitochondria for autophagy through interaction with p62 [138]. In addition, p62 serves as a scaffold for the recruitment of the Ulk1 complex, the core autophagy machinery, through interaction with the FIP200 claw domain, thereby promoting autophagosome formation at ubiquitin condensates containing damaged mitochondrial proteins [184].

#### Ubiquitin-independent or receptor-mediated mitophagy

Mitophagy can also be mediated by mitochondrial integral membrane proteins that have LIRs independently of ubiquitination [132]. Upon mitochondrial depolarization, these receptors accumulate on the OMM and trigger the formation of an isolation membrane around the damaged mitochondria by interacting with Atg8 family proteins (LC3A/B/C, GABARAP, GABARAP-L1/2) on the autophagosome [132].

- (i) BNIP3 and BNIP3L/NIX
  BCL2 interacting protein 3 (BNIP3) is involved in mitochondrial turnover during hypoxia [218]. In response to hypoxia, BNIP3 is upregulated and anchored to the OMM through its C-terminal transmembrane domain. The N-terminal domain carrying the LIR motif is then exposed to the cytosol, allowing it to serve as a mitophagy receptor [54, 80, 146]. Phosphorylation of Ser17 and Ser24 proximal to the LIR region of BNIP3 is critical for LC3 interaction [231]. BNIP3 is upregulated by HIF-1α, thereby mediating mitophagy in response to hypoxia in murine embryonic fibroblast (MEF) cells [217]. BNIP3 is also upregulated by p53, thereby mediating mitochondrial dysfunction and autophagic cell death, the major known function of p53 in the heart [191]. Thus, BNIP3 mediates both adaptive and maladaptive mitophagy in a context-dependent manner.
  BNIP3L/NIP3-like protein X (NIX) is 53–56% homologous to BNIP3 and mediates mitochondrial clearance via programmed mitophagy during reticulocyte maturation and erythrocyte differentiation [154]. NIX promotes mitophagy by recruiting GABARAP-L1 in CCCP-treated cells [129]. The NIX-LC3 interaction is stabilized by phosphorylation of NIX at Ser34 and Ser35 near the LIR motif [147]. As with BNIP3, the C-terminal region of NIX is recruited and homodimerizes for efficient mitophagy initiation [101]. Reactive oxygen species (ROS) accumulation caused by oxidative phosphorylation promotes the recruitment of Rheb (a small GTPase of the Ras superfamily) together with NIX and LC3 to promote mitophagosome formation [106]. In cardiac progenitor cells, mitophagy mediated by BNIP3/NIX facilitates their proper differentiation into myocytes through mitochondrial network reorganization and promotes their survival in the infarcted heart [84].
  How do BNIP3/NIX mediate mitophagy? Several studies have shown that BNIP3 and NIX can activate PINK1–Parkin-mediated mitophagy. Parkin ubiquitinylates NIX and promotes the recruitment of the selective autophagy adaptor NBR1, which can interconnect ubiquitin and LC3/GABARAP to promote autophagosome formation around mitochondria [43]. Additionally, BNIP3 interacts with PINK1 and promotes its accumulation on the OMM, leading to Parkin recruitment to mitochondria [223]. NIX similarly promotes Parkin recruitment to depolarized mitochondria [31]. However, BNIP3 is also involved in Parkin-independent mitophagy. Myeloid cell leukemia-1 (Mcl-1), an anti-apoptotic Bcl-2 member, interacts with BNIP3 to enhance mitophagy in the heart in the presence of energy stress and mitochondrial damage [113]. Mcl-1 has an LIR motif and acts as an adaptor for BNIP3 to promote mitophagy. Since Mcl-1 inhibits general autophagy through interaction with Beclin 1, it could serve as a mechanism that differentially regulates general autophagy and mitophagy.
  BNIP3 may also induce mitophagy through Drp1. As we discuss separately below, Drp1 is a GTPase involved in mitochondrial fission. BNIP3 induces mitochondrial translocation of Drp1, allowing Drp1 to mediate mitochondrial fission and mitophagy in cardiomyocytes [88]. It should be noted that the effect of BNIP3 upon Drp1 is complex and may be detrimental under some conditions through pathological activation of mitochondrial fission and mitophagy. For example, doxorubicin-induced cell death and mitochondrial fission are prevented by downregulation of BNIP3 [29]. A polyphenolic compound, ellagic acid (EA), suppresses mitochondrial injury and necrotic cell death of cardiomyocytes through inhibition of BNIP3 (and subsequent activation of pathological mitophagy), and is thus of therapeutic benefit in lowering oxidative injury and cardiac dysfunction in cancer patients undergoing chemotherapy or under ischemic cardiac stress [29].
  Increasing lines of evidence suggest that the level of BNIP3/NIX is regulated by posttranslational mechanisms. For example, SCF-FBXL4 ubiquitin ligase regulates BNIP3 and NIX localization on mitochondria to preserve normal mitochondria levels. The loss of SCF-FBXL4 leads to excessive mitophagy and perinatal lethality [12]. The UBXD8 adaptor regulates mitophagy by aiding in the recruitment of the hexameric AAA-ATPase valosin-containing protein (VCP) to mitochondria and promoting the degradation of BNIP3 through interaction with ubiquitin E3 ligases in mitochondria [226]. In addition, the mitochondrial protein TMEM11 forms a complex with BNIP3 and BNIP3L and is co-enriched at sites of mitophagosome formation. Lack of TMEM11 hyperactivates mitophagy under normoxic and hypoxia-mimetic conditions, suggesting that it provides a spatial restriction effect on mitophagy activation by BNIP3/BNIP3L [206].
- (ii) FUNDC1
  FUN14 domain-containing protein 1 (FUNDC1) is an integral OMM protein with an N-terminal LIR motif that acts as a receptor for hypoxia-induced mitophagy [95]. FUNDC1 is regulated via phosphorylation and dephosphorylation events on Ser13 and Tyr18 near its LIR motif. Normoxic conditions favor Ser13 and Tyr18 phosphorylation of FUNDC1 by casein kinase 2 (CK2) and Src tyrosine kinase, respectively, to negatively regulate FUNDC1 interaction with LC3 [15, 95]. Under hypoxia, Src kinase is inactivated and the FUNDC1–LC3 interaction is stabilized by decreasing Tyr18 phosphorylation. Similarly, removal of Ser13 phosphorylation by PGAM5, a serine/threonine phosphatase, promotes mitophagy [15]. However, the PGAM5–FUNDC1 interaction is prevented by BCL2L1/Bcl-xL (anti-apoptotic BH3 domain-containing molecule) under normoxic conditions [197]. On the other hand, in the presence of hypoxia or mitochondrial depolarization, phosphorylation of Ser17 of FUNDC1 by Ulk1 recruited to fragmented mitochondria promotes mitophagy by increasing interaction with LC3 [198].These studies emphasize the importance of FUNDC1 as a mitophagy receptor under hypoxic stress conditions. FUNDC1-mediated mitophagy also promotes cardiac progenitor cell differentiation and survival in the infarcted heart [84]. Hypoxia acclimation with a low-pressure hypoxic animal chamber alleviated cardiac dysfunction and fibrosis caused by MI injury in mice through activation of FUNDC1-mediated mitophagy [92]. The role of FUNDC1 in ischemia/reperfusion injury in the heart is discussed further later in this review.
- (iii) BCL2L13﻿
  BCL2L13 is a single pass membrane protein anchored on the OMM and contains two LIR motifs. It regulates mitochondrial morphology such that the fragmented state is increased when it is overexpressed and the elongated state is increased when it is knocked down [116]. Involvement of BCL2L13 in inducing mitophagy was first shown in yeast studies, in which BCL2L13 restored mitophagy in yeast cells lacking Atg32, a mitophagy receptor [116]. BCL2L13-dependent mitophagy was shown to be mediated through conventional Atg7 via Atg8 lipidation in Atg32-lacking yeast cells [116]. Specifically, a mutation in one of the LIRs of BCL2L13 inhibited mitophagy in yeast cells lacking Atg32, suggesting the involvement of Atg8 in BCL2L13-mediated mitophagy [116], and phosphorylation at Ser272 near the second LIR motif is important for the BCL2L13–LC3 interaction [116]. In HEK293 and HeLa cells, BCL2L13 recruits the Ulk1 complex (including FIP200, Atg13 and Atg101) along with LC3B to autophagosomes to induce mitophagy under starvation conditions [116].
- (iv) FKBP8
  FK506-binding protein 8 (FKBP8) is an OMM integral protein with an N-terminal LIR motif and a C-terminal transmembrane domain. FKBP8 preferentially binds to LC3A over other Atg8 family proteins in vivo and mediates mitophagy in HeLa cells [9, 94].
- (v) Prohibitin 2
  Prohibitins (PHB) are IMM proteins. PHB2 is exposed to the cytosol in the presence of mitochondrial membrane depolarization and increased proteasomal activity that ruptures the OMM [194]. PHB2 associates with LC3 through its LIR domain and the p62 adaptor to target mitochondria for autophagic degradation [200]. PHB2 also mediates Parkin-induced mitophagy under energetic stress conditions in HeLa cells and MEFs [194].
- (vi) Cardiolipin
  Cardiolipin is a phospholipid in the IMM that is exposed to the cytosol only when the OMM is disrupted by mitochondrial damage and elicits autophagosome formation around damaged mitochondria via its interaction with LC3 [21, 158]. Mesenchymal stem cells (MSCs) derived from high-fat diet (HFD)-induced obese mice (MSC-Ob) have a reduced cardiolipin content and less ability to clear damaged mitochondria compared to MSCs from control mice fed a normal diet [151].
- (vii) AMBRA1
  Autophagy/Beclin 1 regulator 1 (AMBRA1) is an adapter protein in the autophagy signaling network. AMBRA1 is involved in the early steps of autophagosome core complex formation in mTORC1-dependent autophagy [22]. Under basal conditions, AMBRA1 is present in mitochondria and its pro-autophagic activity is inhibited by Bcl-2. However, AMBRA1 directly interacts with LC3 through its LIR motif to recruit mitochondria to autophagosomes upon mitophagy induction. This interaction is crucial for regulating both canonical Parkin-dependent and -independent mitochondrial clearance in fibroblasts of human and PINK1^−/−^ mouse origin, as well as in HEK293 and HeLa cell lines [166]**.**
- (viii) ATAD3B
  ATPase family AAA domain-containing protein 3B (ATAD3B), which is only expressed in primates, is a mitochondrial membrane-bound ATPase that was recently identified as a mitophagy receptor [161]. The ATAD3B protein has an LIR motif that binds to LC3 and stimulates oxidative stress-induced mitophagy without the assistance of PINK1, facilitating the removal of oxidative stress-damaged mtDNA. When ATAD3B and ATAD3A hetero-oligomerize under normal conditions, ATAD3B's C-terminal region is directed toward the mitochondrial intermembrane space, preventing mitophagy. Decreased ATAD3B–ATAD3A hetero-oligomerization as a result of oxidative stress-induced mtDNA damage or mtDNA depletion causes exposure of the ATAD3B C-terminus at the OMM, which then attracts LC3 to stimulate mitophagy [161].
- (ix) Choline dehydrogenase (CHDH)
  CHDH catalyzes the dehydrogenation of choline to betaine aldehyde in mitochondria and participates in glycine, serine, and threonine metabolism. Under normal circumstances, CHDH is found in both the IMM and OMM. When the mitochondrial membrane potential is disrupted, CHDH builds up on the OMM and interacts with p62 via its Phox and Bem1 (PB1) domain, forming a CHDH-p62-LC3 complex and mediating mitophagy [136].

#### Alternative mitophagy and mechanism

The forms of mitophagy discussed above are all mediated through autophagosomes associated with LC3, requiring the Ubl conjugation system. However, some forms of mitophagy occur independently of the conventional autophagy mechanisms. Using Atg5 knockout cells, Nishida et al. discovered an Atg5- and Atg7-independent mechanism of autophagy, referred to as alternative autophagy [127]. The two Ubl conjugation systems, which involve Atg5, Atg7, and LC3, are not utilized by alternative autophagy. Like conventional autophagy, alternative autophagy utilizes double-membrane autophagosomes to internalize cargo for degradation via fusion with lysosomes. However, autophagosomes in alternative autophagy are produced through a Rab9-dependent, but LC3-independent, mechanism [127].

Although both Ulk1 and Ulk2 help initiate conventional autophagy, Ulk2 function is thought to be redundant, whereas the loss of Ulk1 typically causes disruption of autophagy [56, 216]. Furthermore, Ulk1 is a critical initiator of both the conventional and alternative processes, in contrast to Ulk2, which is only involved in conventional autophagy [83]. Both conventional and alternative autophagy are regulated by phosphorylation of Ulk1 by mTORC1 (at Ser757, inhibition) and AMPK (at Ser317 and Ser777, activation) [75], and Beclin 1 and Vps34 are among the autophagy-related targets that are phosphorylated by Ulk1 [36]. On the other hand, in Atg5-deficient MEF cells treated with etoposide, the necroptosis-regulating kinase RIPK3 interacts with and phosphorylates Ulk1 at Ser746. RIPK3-dependent phosphorylation of Ulk1 at Ser746 only activates alternative autophagy, without activating necroptosis or conventional autophagy, indicating a clear functional distinction between conventional and alternative mechanisms [183, 195].

In alternative autophagy, autophagosomes originate from the trans-Golgi network [127]. At the isolation membrane, WIPI family proteins perform a crucial PIP3 effector function [115]. WIPI3, rather than WIPI1 and WIPI2, is essential for producing alternative isolation membranes [207]. Additionally, a recent study found that Golgi-resident Rab2 participates in the formation of autophagosomes by separating from the Golgi apparatus under autophagy-inducing conditions to interact with Ulk1 [32]. Ulk1-deficient mice are viable, in contrast to mice with knockouts of various important Atg genes, such as Atg5, Atg7, and Atg12, which are fatal to neonates [82]. However, Ulk1-knockout mice exhibit defects in mitochondrial autophagy in primary hepatocytes during erythrocyte development [83, 157].

**Fig. 2:**
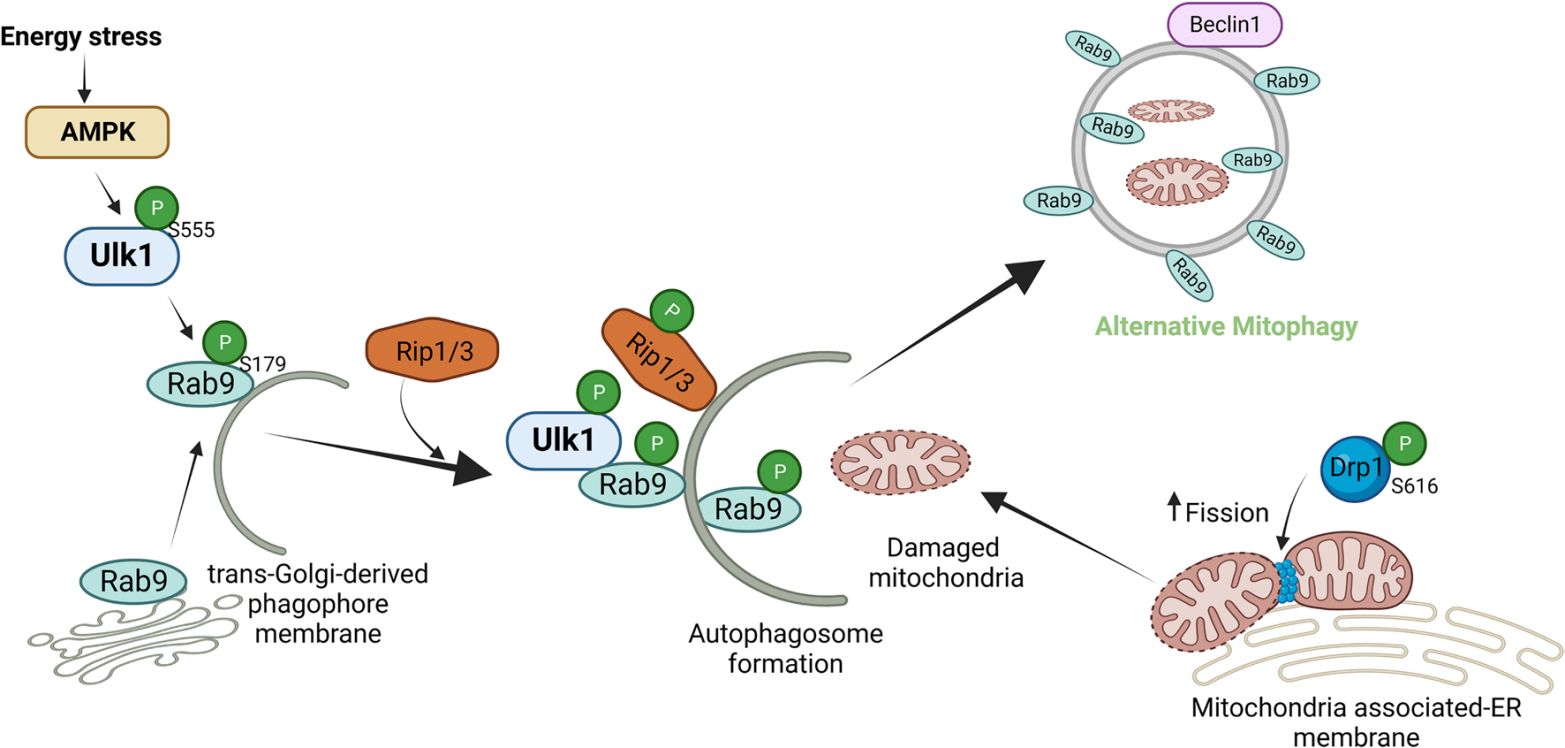
Mechanism of alternative mitophagy. Energy stress activates AMPK-mediated phosphorylation of Ulk1 at Ser555. The activated Ulk1 phosphorylates Rab9 at Ser179 and initiates phagophore formation utilizing trans-Golgi-derived membrane. Activated Ulk1-Rab9 recruits Rip1/3 kinase and phosphorylates Drp1 at Ser616, which activates mitochondrial fission to segregate damaged mitochondria and promote alternative mitophagy at the mitochondrial-associated ER membrane. The figure was created with Biorender.com

There is a form of mitophagy that uses a molecular mechanism similar to that of alternative autophagy and is, thus, termed alternative mitophagy. Alternative mitophagy can be observed even when conventional autophagy/mitophagy is inhibited, such as in the presence of Atg7 downregulation [120, 152, 180]. In alternative mitophagy, damaged mitochondria are sequestrated in autophagosomes associated with Rab9, but not LC3, through Ulk1-dependent mechanisms. Although depolarized mitochondria can be found in the cargo, how damaged mitochondria are tagged for degradation remains unclear. What is known, however, is that alternative mitophagy is associated with the formation of a large protein complex consisting of Ulk1-Rab9-Rip1-Drp1 near mitochondria-associated endoplasmic reticulum membrane (MAM) [179]. Drp1 is a conserved dynamin GTPase superfamily protein required for mitochondrial fission and various forms of mitophagy, including Parkin-dependent, Parkin-independent, receptor-mediated, and alternative mitophagy [69, 182]. Multiple kinases, such as Cdk1, Erk2, and Rip1, phosphorylate Drp1 at Ser616 and enhance fission [66, 71, 118, 152, 199]. Under stress conditions, Ulk1 interacts with Rab9 and phosphorylates it at Ser179, which is a crucial first step for the formation of the Ulk1-Rab9-Rip1-Drp1 complex [152]. A signaling complex comprising Rip1 and Drp1 is formed in response to Ulk1 activation and phosphorylation of Rab9. Rip1-mediated Drp1 phosphorylation at Ser616 then promotes mitochondrial fission, and Rab9-mediated autophagosomes form around damaged mitochondria [152]. A schematic representation of the molecular mechanisms involved in alternative mitophagy is given in Fig. 2. As mentioned above, the materials for generating alternative autophagosomes are derived from trans-Golgi membranes. Alternative mitophagy is activated after conventional mitophagy is inactivated in diabetic hearts [179, 180]. Thus, inactivation of conventional mitophagy may induce some of the mechanisms that stimulate alternative mitophagy.

Other processes also degrade damaged mitochondria, using some of the same molecules as those utilized by conventional mitophagy but via distinct mechanisms. For example, damaged mitochondria with OMM proteins ubiquitinated by Parkin are also sequestrated by Rab5-dependent early endosomes via the ESCRT machinery and degraded in lysosomes [53]. The endosomal-mediated degradation of mitochondria is initiated by the same mechanism as Parkin-mediated mitophagy and requires Beclin 1 [53], suggesting that crosstalk may exist between mitophagy and the endosomal pathway. More investigation is needed to clarify the functional relevance of endosomal-mediated mitochondrial clearance in the heart in vivo.

#### Micromitophagy

Microautophagy is a mechanism through which cytosolic materials are directly engulfed by lysosomes through membrane invagination. During ischemia–reperfusion (I/R) in the heart, damaged mitochondria can be taken up by lysosomes directly. I/R promotes the association of GAPDH with mitochondria and directs the uptake of damaged mitochondria into multiorganellar lysosomal-like (LL) structures for elimination [209]. Protein kinase Cδ (PKCδ) inhibits this process through phosphorylation of GAPDH at Thr246, thereby leading to accumulation of damaged mitochondria at the edge of the LL structure and causing apoptosis. Inhibition of PKCδ or expression of a phosphorylation resistant GAPDH mutant during I/R rescues mitophagy, promotes clearance of damaged mitochondria, and downregulates apoptosis [209].

MDVs represent another form of microautophagy, in which vesicles enriched in mitochondrial proteins bud off from mitochondria, transit into multivesicular bodies and are engulfed by lysosomes [90, 104, 165]. MDVs are stimulated as an early response to oxidative stress and deliver dysfunctional proteins and lipids from mitochondria as cargo to lysosomes without the involvement of mitochondrial depolarization and fission machinery [104, 165]. Although the process is independent of the macroautophagy machinery, including Atg5 and LC3, it requires PINK1 and Parkin [104, 165].

Taken together, mitochondria can be degraded through multiple mechanisms. How each mechanism of mitochondrial degradation is regulated by stress and whether the activities of the different mechanisms of mitochondrial degradation affect one another remain to be clarified.

#### Autophagic secretion of mitochondria

Heterophagy is a mechanism whereby damaged mitochondria are ejected from cells and degraded by macrophages [126]. This type of transcellular mitochondrial degradation system exists in the heart, where damaged mitochondria are packed in exophores, large membrane-surrounded vesicles, which are then phagocytized and degraded by resident macrophages after release into the extracellular space [125]. Not only are exophores LC3( +), but the release of exophores is affected by the level of autophagy, suggesting that the exophore-mediated elimination of damaged mitochondria is controlled by mechanisms shared with autophagy. Suppression of exophore phagocytosis or ablation of resident macrophages induces inflammation. Thus, removal of damaged mitochondria through LC3( +) exophores is a mitochondrial quality control mechanism [126]. A recent report showed that damaged mitochondria sequestered into vesicles through PINK1–Parkin-mediated mitophagy are also secreted into the extracellular space, especially when the mAtg8 conjugation system, including Atg3, Atg5, and Atg7, is not functional [172]. Importantly, secreted mitochondria elicit innate immune responses through systemic activation of the cGAS-STING pathway [172]. This highlights the importance of the mAtg8 lipidation system in assuring the completion of mitophagy via lysosomal degradation without activation of inflammatory responses. Thus, it remains to be clarified whether secretion of damaged mitochondria initiated by the PINK1–Parkin-dependent mechanism of mitophagy can play a salutary role in maintaining mitochondrial quality. In addition, cells, including cardiomyocytes, can transfer intact mitochondria from one cell to another, as a cell protective mechanism [39, 64]. The transfer is mediated through several different mechanisms, including tunneling nanotubes and extracellular vesicles [39]. Since extracellular vesicles and MDVs share some properties and the release of extracellular vesicles is affected by lysosomal activity, it is possible that the release of intact mitochondria and eventual improvement of mitochondrial quality control in recipient cells are also controlled by autophagy and mitophagy in host cells. Further investigation is required to clarify this issue.

### Mitochondrial fission and fusion

Mitochondria are highly dynamic organelles that constantly undergo fusion and fission to maintain homeostasis in response to changes in the cellular environment. Mitochondrial fusion is coordinated by Mfn1, Mfn2, and optic atrophy 1 (OPA1), whereas fission is regulated by Drp1 [119, 196]. In general, elongated mitochondria are protected from degradation by mitophagy, carry more cristae, and maintain ATP production through increased activity of ATP synthase [49]. Furthermore, mitochondrial fusion rescues a part of mitochondria from reversible damage [185]. On the other hand, isolation of severely damaged mitochondria through fission can protect the healthy portion of mitochondria by preventing the spread of depolarization and ROS [211]. In addition, physiological mitochondrial fragmentation mediated by Drp1 is important to meet the energetic demands during exercise [25]. Thus, understanding the physiological function of fusion and fission is important.

Mfn1 and Mfn2 are specialized proteins localized on the OMM that regulate the fusion of the OMM. They are dynamin-like GTPases that contain N-terminal catalytic GTP-binding domains and C-terminal transmembrane domains [189]. The C-terminal transmembrane domains anchor Mfn1/2 to the OMM, where they interact with adjacent mitochondria via a heptad repeat region [78, 188]. Since individual overexpression of Mfn1 or Mfn2 can rescue the loss of the other protein to promote fusion, Mfn1/2 presumably have redundant functions [16]. However, genetic mutation of Mfn2 was shown to interrupt mitochondrial fusion, leading to a neurodegenerative condition [40]. Following myocardial IR injury, matrix metalloproteinase-2 (MMP-2) activation and cleavage of Mfn2 leads to impaired myocardial contractile function, lowered mitochondrial respiration, and elevated inflammasome response [7]. MORN repeat-containing protein 4 (MORN4) directly binds to Mfn2 and promotes phosphorylation of Mfn2 at Ser442 by Rho-associated protein kinase 2 (ROCK2) to induce mitochondrial dynamics and mitophagy [229]. Downregulation MORN4 during myocardial ischemia accelerated cardiac injury and fibrosis, exacerbating cardiac dysfunction in a murine model [229].

Fusion of the IMM is regulated by OPA1, a dynamin-like GTPase that is localized on the IMM [28] and also regulates crista formation and maintenance. OPA1 is processed to a membrane-bound long isoform (L-OPA1) by mitochondrial processing peptidase (MPP). However, L-OPA1 can also be proteolytically cleaved to a short isoform (S-OPA1) by two IMM peptidases, overlapping proteolytic activity with m-AAA protease 1 (OMA1) and YME1 Like 1 ATPase (YME1L1) [5]. The balance between L-OPA1 and S-OPA1 is regulated by YME1L and OMA1 under basal conditions. When mitochondrial stress occurs, the activity of OMA1 is increased and L-OPA1 is actively cleaved to S-OPA1. The resulting imbalance between L-OPA1 and S-OPA1 promotes mitochondrial fission [99, 188]. Upregulation of OPA1 at appropriate levels protects the heart against ischemia [23, 170, 186]. Furthermore, inhibition of OMA1 protects the heart against heart failure in response to multiple types of cardiac insult [2] by preventing crista remodeling. These findings suggest that OPA1 and inhibition of OMA1 play salutary roles during heart failure, primarily by preventing crista remodeling. However, how degradation of irreversibly damaged mitochondria is affected by these interventions remains unknown.

OMM constriction occurs at mitochondria–ER contact sites where Drp1 is oligomerized. Drp1 is a cytosolic protein with an N-terminal GTPase domain, a middle domain, a variable domain, and a C-terminal GTPase effector domain [142]. Drp1 is recruited to sites of mitochondrial fission in a phosphorylation-dependent manner, through interactions with mitochondrial OMM proteins such as Fis1, MFF, and mitochondrial dynamics proteins of 49 and 51 kDa (MiD49, MiD51) [96, 210]. Fis1 was initially proposed as a Drp1 receptor in yeast, but the role of mammalian Fis1 is less clear [114]. While it may not be required for Drp1 recruitment to the OMM, as shown in Fis1-null MEFs, overexpression of human Fis1 can induce mitochondrial fragmentation in the absence of Drp1 [96, 212]. Fis1 activates mitochondrial fission and suppresses fusion by inhibiting the GTPase activities of fusion proteins Mfn1, Mfn2, and OPA1 [212]. While OMM constriction has been studied extensively, the mechanism of IMM division remains elusive. Recent studies have shown that an intra-mitochondrial influx of Ca^2+^ activates IMM constriction at mitochondria–ER contact sites [14, 19], but further studies are required to fully understand the fission process of the IMM. A recent study using yeast and HeLa cells showed that mitofissin (Atg44), a mitochondrial intermembrane space protein, mediates fission and contributes to mitophagy [42]. Mitofissin is required for the formation of mitochondrial protrusions that are eventually fragmented and encapsulated by phagosomes for clearance [42].

Mitochondrial fission allows the segregation of unhealthy mitochondria so that they can be eliminated by mitophagy [185]. Although direct involvement of Drp1 in the mitophagy machinery has been shown in yeast [1, 70, 100], the mechanism by which mitochondrial fission is coupled with mitophagy has not been fully elucidated in mammalian cells [65, 69, 164]. Separation of damaged mitochondria during mitophagy takes place even in the absence of Drp1 in yeast and HeLa cells, where the damaged portion of mitochondria can be separated by autophagosomal constriction [208]. However, the loss of Drp1 function does inhibit mitophagy in the heart under some conditions [182]. When Drp1 is phosphorylated at Ser616, it is localized at the MAM, where mitochondrial fission takes place in cardiomyocytes. As noted above, Drp1 associates with a large protein complex at the MAM, thereby mediating alternative mitophagy in cardiomyocytes during ischemia and HFD consumption [179]. It should be noted that Drp1 can regulate mitophagy even without physical contact with the damaged mitochondria. For example, Drp1 positively mediates autophagosome formation by alleviating the inhibition of Beclin 1 by Bcl-2/Bcl-xL [182]. Furthermore, Drp1 also regulates mitochondrial function through fission-independent mechanisms [219]. Thus, it is possible that the effect of loss of Drp1 function upon mitophagy could be secondary to its effect upon global changes in mitochondrial function. Indeed, although Drp1 promotes physiological mitochondrial fragmentation during exercise, mitophagy is inhibited [25]. Although this makes practical sense, in that the mitochondria are not depolarized and it would be undesirable for the content to be decreased when coping with a high energy demand, the mechanism by which Drp1 actively inhibits mitophagy is unknown.

### The role of mitophagy during cardiac stress

Here we discuss activation of mitophagy during cardiac stress and its functional significance. We will limit our discussion to three representative stress conditions in which activation of mitophagy plays a critical role in regulating the survival and function of cardiomyocytes.

#### Mitophagy in ischemia and reperfusion (I/R)

While reperfusion after ischemia is critical for myocardial survival, it dramatically increases oxidative damage through generation of ROS, causing myocardial cell death [20, 58, 117]. Mitochondrial ROS trigger the opening of the mitochondrial permeability transition pore (mPTP) on the IMM [20]. mPTP opening during I/R prevents the recovery of ATP production and causes cardiomyocyte death through necrosis [52].

In general, mitophagy is activated in the heart and plays a protective role during I/R by alleviating the insults from damaged mitochondria and promoting mitochondrial biogenesis for recovery [81]. For example, phosphoglycerate mutase family member 5 (PGAM5) is crucial for mitochondrial homeostasis, promoting mitophagy through stabilization of PINK1; PGAM5 knockout mice showed inhibited PINK1-dependent mitophagy in the heart, resulting in the accumulation of damaged mitochondria, increased oxidative stress, and exacerbated necroptosis during I/R [97]. The mitochondrial Zn^2+^ transporter (ZIP7 or Slc39a7) is upregulated during the reperfusion phase and negatively regulates mitophagy, contributing to increased reperfusion injury [220]. ZIP7 levels are high in the mitochondria of cardiac tissue from heart failure patients [220]. Cardiac-specific downregulation of ZIP7 decreased ROS production, enhanced mitophagy, and reduced infarct size, conferring protection against reperfusion injury [220]. Transient receptor potential mucolipin 1 (TRPML1) is activated secondary to increased ROS after I/R, thereby inducing lysosomal zinc release into the cytosol and ultimately blocking autophagy in cardiomyocytes, possibly by disrupting fusion between autophagosomes and lysosomes [167, 202]. Pharmacological and genetic inhibition of TRPML1 channels effectively reduced infarct size and rescued heart function in mice subjected to I/R in vivo by restoring impaired myocardial autophagy and reducing accumulation of damaged mitochondria [167, 202]. Downregulation of endogenous Drp1 inhibits mitophagy and increases cardiomyocyte death during I/R [34]. CLOCK, a circadian gene, transcriptionally coordinates genes involved in mitochondrial fission and fusion and mitophagy, thereby promoting cell survival [145]. Thus, interventions that stimulate the direct regulators of mitophagy in cardiomyocytes may alleviate I/R injury by preconditioning the heart and promoting mitochondrial quality control mechanisms. Mitophagy is also activated in platelets during I/R. A synthetic peptide inhibiting the FUNDC1-LC3 interaction inhibits mitophagy in platelets during hypoxia and prevents preconditioning of the heart against reperfusion injury [224]. Thus, interventions that stimulate mitophagy in non-myocyte populations may also be effective in reducing I/R injury. However, caution must be exercised in utilizing these interventions since many molecules have multiple cellular functions and their effects are context dependent. For example, the effect of Mdivi-1, a chemical inhibitor of Drp1, upon I/R injury is dose and context dependent [148, 222]. Myocardial ischemia without reperfusion activates not only conventional mitophagy but also alternative mitophagy in the heart [152]. Although suppression of conventional mitophagy did not increase myocardial damage after 3 h of ischemia, suppression of alternative mitophagy with Rab9(S179A) significantly exacerbated myocardial damage, suggesting that alternative mitophagy may play a more prominent protective role than conventional mitophagy during prolonged ischemia in the heart [152]. The role of alternative mitophagy during reperfusion remains to be elucidated.

Mitophagy during I/R is also negatively regulated by upstream signaling molecules. Casein kinase 2α (CK2α), a constitutive serine/threonine kinase, suppresses FUNDC1 by phosphorylating it at Ser13 [8, 15]. CK2α protein expression is upregulated during acute cardiac I/R injury and inhibits FUNDC1-dependent mitophagy [228]. Cardiac-specific CK2α knockout in mice abrogated cardiac dysfunction after I/R injury [228]. Similarly, genetic deletion of mammalian STE20-like kinase 1 (Mst1), a potent inhibitor of autophagy, activated FUNDC1-induced mitophagy and reduced cardiomyocyte mitochondrial apoptosis, resulting in protected cardiac function [214]. Thus, endogenous Mst1 also inhibits FUNDC1-mediated mitophagy, leading to increased cardiac injury [214]. Myocardial ischemia induces accumulation of CO_2_ and bicarbonate, the latter of which, in turn, suppresses mitophagy during reperfusion, resulting in exacerbated myocardial injury [143]. Thus, an alternative strategy for protecting the heart against I/R injury could be to inhibit the negative regulators of mitophagy.

It should be noted that some reports showed that mitophagy can be over-activated during I/R and, thus, downregulation of mitophagy may mitigate myocardial injury under some conditions. For example, downregulation of mitophagy during I/R via overexpression of RNA methylation reading protein YTHDF2 relieves myocardial I/R injury by downregulating BNIP3 mRNA expression in hypoxia–reperfusion (H/R)-treated H9c2 cells or myocardial I/R injury (MIRI) rats [11]. Notch1 suppresses the PTEN-PINK1-Parkin signaling and downregulates mitochondrial fusion/fission and mitochondrial autophagy, conferring protection against I/R injury in cardiomyocytes and a rat I/R injury model [203]. In I/R rat models, exercise-induced parasympathetic nerve function increased myocardial M2 acetylcholine receptor (M_2_AChR) protein expression and effectively reduced mitophagy, endoplasmic reticulum stress (ERS), and apoptosis [17].

We and others have shown that autophagy can become dysregulated during the late stages of I/R injury and that excessive autophagy induces cell death. One mechanism mediating cell death induced by dysregulated autophagy is autosis, a unique form of cell death characterized by specific morphological and biochemical features [121]. Upregulation of Rubicon during the late stage of I/R blocks autophagic flux, thereby inducing excessive accumulation of autophagosomes and dysfunctional intracellular organelles. It is currently unknown whether dysregulation of mitophagy induces autosis. Since, in theory, mitophagy selectively eliminates already damaged mitochondria, it may not participate in autosis. However, further investigation is required to determine whether excessive degradation of mitochondria can take place during I/R through non-selective destruction due to dysregulated autophagy.

**Fig. 3:**
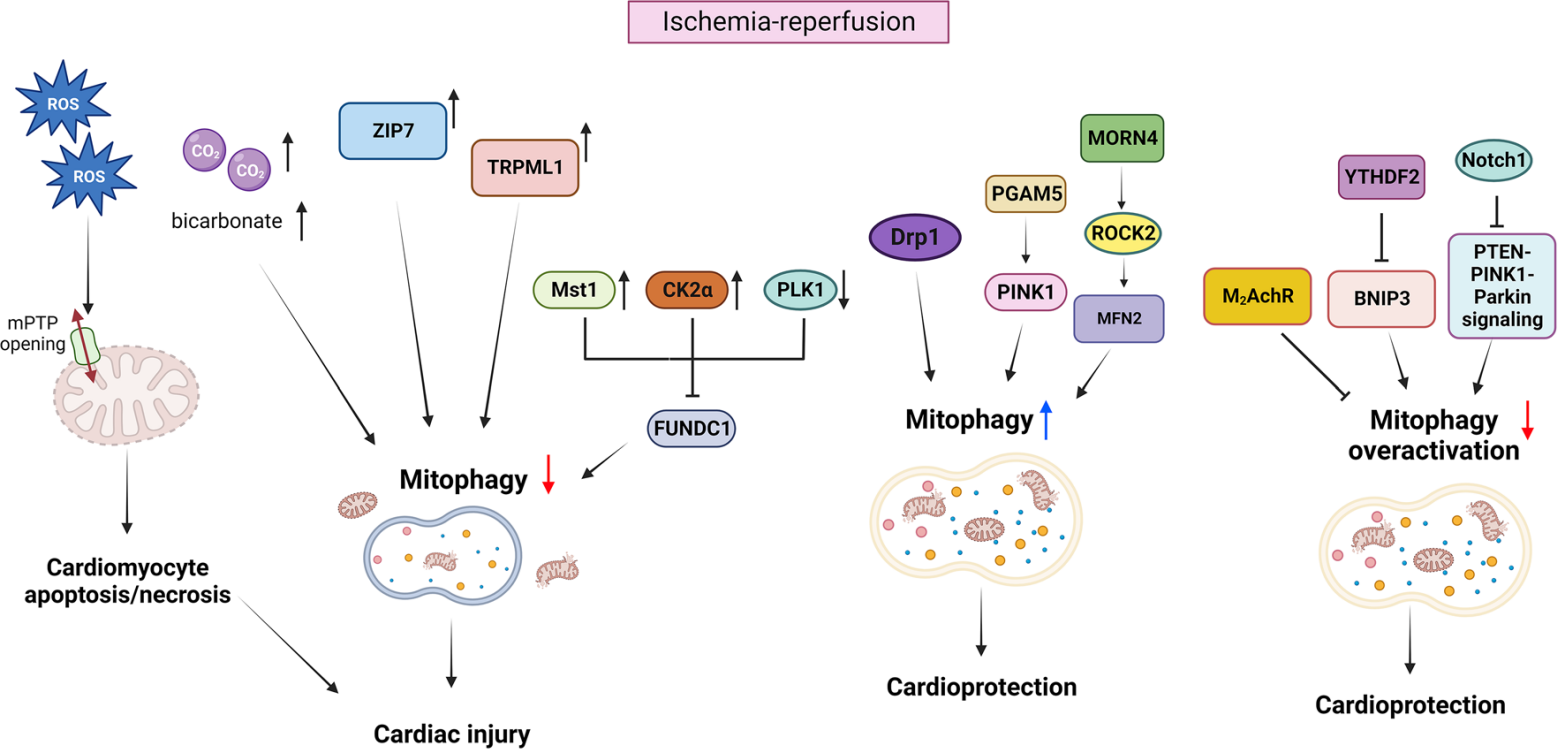
Mitophagy in ischemia–reperfusion injury. During myocardial reperfusion following ischemic injury, increased ROS production causes mPTP opening, thereby leading to cardiomyocyte apoptosis and necrosis. In this condition, accumulated CO_2_ and bicarbonate inhibit mitophagy and promote myocardial injury. ZIP7 and TRPML1 upregulation during I/R inhibits mitophagy thereby contributing to increased reperfusion injury. Upregulated Mst1 and CK2α or downregulated PLK1 inhibit FUNDC1-mediated mitophagy, resulting in exacerbated cardiac injury. Mst1 may inhibit mitophagy through Beclin 1 phosphorylation and inhibition of autophagosome formation. Drp1-mediated and PGAM5-PINK1-mediated mitophagy protects the heart against I/R injury. MORN4 promotes ROCK2-mediated phosphorylation and activation of MFN2 leading to increased mitochondrial dynamics and mitophagy to enhance cardioprotection during I/R injury. Inhibition of overactivation of mitophagy during I/R injury is cardioprotective in certain conditions. M_2_AChR signaling inhibits excessive mitophagy. Notch1 inhibits PTEN-PINK1-Parkin signaling-mediated mitophagy. YTHDF2 inhibits BNIP3 mRNA expression and downregulates mitophagy. The figure was created with Biorender.com

An important caveat is that what we currently know about the role of mitophagy during I/R is based upon studies conducted with rodents. Studies with large mammals and humans remain sparse. In a porcine model of I/R, pre-treatment with chloramphenicol succinate (CAPS) (an autophagy activator) greatly reduced infarct size and exerted cardioprotection compared to saline treatment [153]. In patients undergoing coronary artery bypass or valve surgery requiring cardiopulmonary bypass (CPB), an increase in autophagic flux during surgery, indicated by decreases in p62 from right atrial appendage biopsies, was inversely correlated with mortality post-surgery, suggesting that autophagy is cardioprotective in humans [67]. However, another study showed that autophagy, indicated by changes in autophagy markers in left ventricular biopsies, is not activated by early reperfusion or remote ischemic preconditioning in patients undergoing coronary artery bypass grafting. Thus, other mechanisms besides autophagy may predominantly mediate cardioprotection during myocardial ischemia and reperfusion in humans [44]. Further investigation with large mammals and humans is essential. A schematic representation of the effect of various factors discussed here on mitophagy and their influence on the heart during I/R injury is shown in Fig. 3.

#### Mitophagy during cardiac hypertrophy and heart failure

Transverse aortic constriction (TAC) is commonly used to model pressure overload-induced heart failure in mice. Hemodynamic forces induce not only mechanical stress but also neurohormonal and autocrine/paracrine responses, culminating in cellular stress at the level of organelles, including the endoplasmic reticulum and mitochondria. Pressure overload rapidly induces autophagy in cardiomyocytes, which plays both protective and detrimental roles in the heart depending upon the extent of stress and the timepoint [122, 149, 155]. LC3-dependent classical autophagy was shown to be activated acutely post-TAC and rapidly reverted to the baseline level by 24 h [159]. Mitophagy was subsequently activated from Day 3 to Day 7 post-TAC, as demonstrated using electron microscopy and Mito-Keima, a fluorescent indicator of mitophagy [120]. Thus, although mitophagy is activated by pressure overload, its activation is transient, and, importantly, heart failure develops after mitophagy is inactivated. Treatment with TAT-Beclin 1 [160], a peptide that allows mobilization of endogenous Beclin 1 from the intracellular storage site, after mitophagy was inactivated partially rescued mitophagy and improved heart function in the presence of pressure overload. These results suggest that mitophagy plays a protective role in the heart during pressure overload [159]. Consistently, activation of mitophagy is generally protective during other forms of heart failure [60, 168, 190, 230]. It is possible that selective autophagy, including mitophagy and ERphagy [109], may be more consistently protective against heart failure than general autophagy.

General autophagy, evaluated with GFP-LC3, and mitophagy, evaluated with Mito-Keima, appear to take place with distinct time courses. The difference in time course between general autophagy and mitophagy was also observed in the heart in response to other forms of stress, including ischemia and HFD consumption [65, 105, 117, 119, 120, 152, 179–182], and was also noted in other cell types [3, 48, 108]. One possibility is that selective forms of autophagy, including mitophagy, are mediated through molecular mechanisms distinct from those of conventional autophagy, like alternative mitophagy. Thus, to reactivate mitophagy in failing hearts, it would be essential to clarify possible time-dependent changes in the underlying mechanism of mitophagy and identify molecular interventions, like TAT-Beclin 1, that effectively reactivate mitophagy. For example, Ulk1/Rab9-mediated alternative mitophagy is activated during pressure overload [120]. Cardiac-specific Ulk1-knockout (Ulk1cKO) mice, in which alternative mitophagy is inhibited, developed cardiac dysfunction as early as 3 days post-TAC, as compared to 2 weeks in wild-type mice [120]. However, rescue of LC3-dependent mitophagy using TAT-Beclin 1 in Ulk1cKO mice improved cardiac function after TAC [120]. This suggests that compensation with a TAT-Beclin 1-activatable form of mitophagy is beneficial under pressure overload conditions. Drp1 was found to translocate from the cytosol to mitochondria and activate mitophagy in the heart under pressure overload [110, 159]. Additionally, cardiac-specific heterozygous Drp1 knockout mice showed exacerbated heart failure in response to pressure overload even after activation of autophagy with TAT-Beclin 1, suggesting that Drp1 is required for mitophagy in the heart in response to pressure overload [110, 159]. In addition, Kruppel-like factor 4 (KLF4), a member of the zinc finger family of transcription factors, directly binds to the promoter of Ulk1/2, molecules implicated in mitophagy during pressure overload stress [93].

Volume overload is observed in the chronic phase of myocardial infarction (MI), which also leads to cardiac hypertrophy, chamber dilation, and heart failure. Parkin has been shown to be essential for mitophagy in cardiomyocytes following MI, with Parkin deficiency leading to exacerbation of cardiac remodeling and heart failure after MI. Cardiomyocytes from Parkin knockout mice exhibit accumulation of damaged mitochondria due to reduced mitophagy [81]. However, PINK1 appears not to be essential for mitophagy after MI since Parkin recruitment to damaged mitochondria was unaffected by the loss of PINK1 [79].

**Fig. 4:**
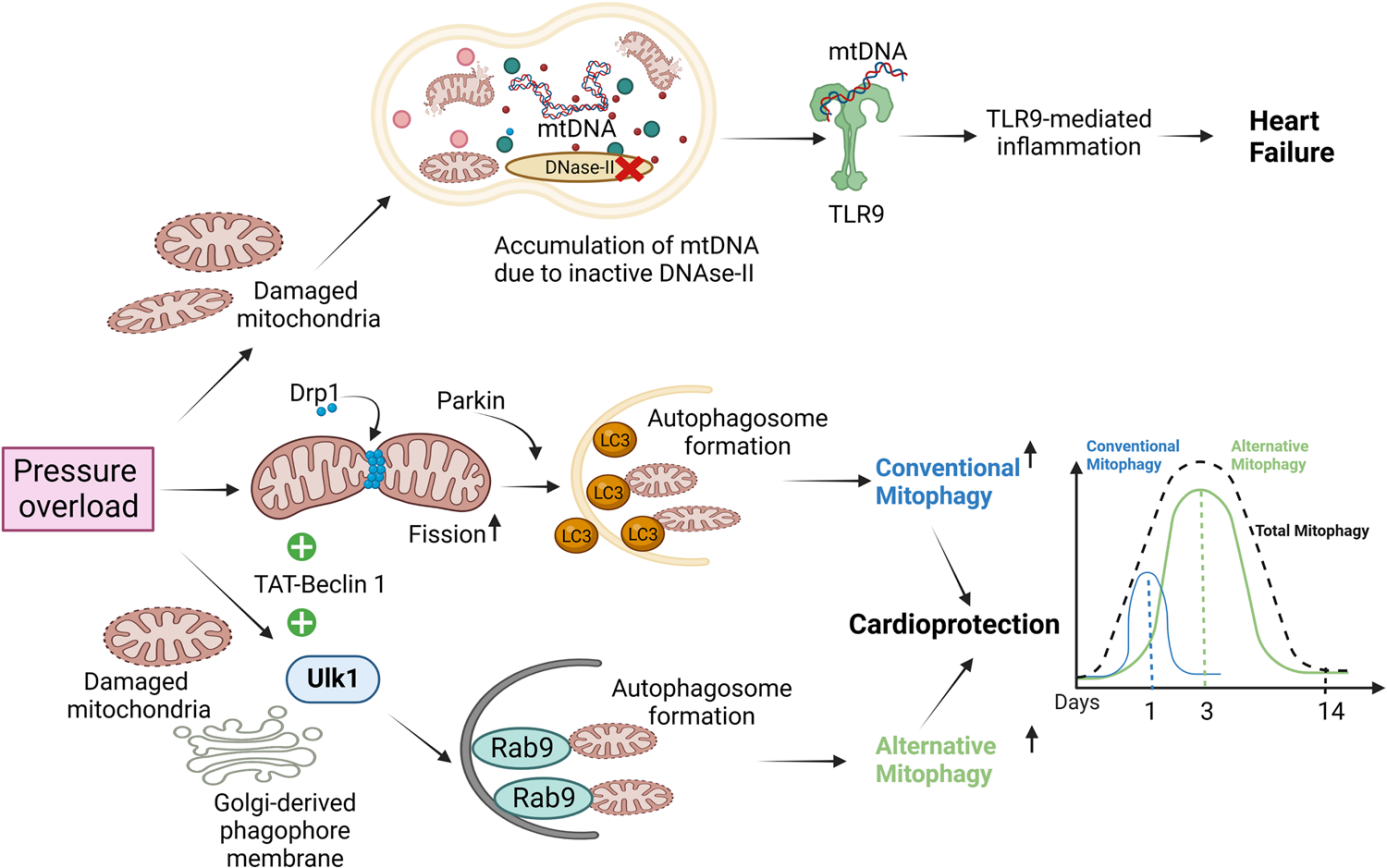
Mitophagy in pressure overload-induced hypertrophy. Transient activation of conventional autophagy during pressure overload occurs one day after TAC, whereas activation of mitophagy occurs thereafter, peaking at around 3 and 7 days after TAC. In particular, mitophagy activation after inactivation of conventional autophagy occurs through an Ulk1-Rab9-dependent alternative mitophagy mechanism. Both autophagy and mitophagy contribute to cardioprotection under pressure overload stress. Pressure overload causes mitochondrial dysfunction and inhibits the action of DNase II in the lysosome, leading to non-degradation of mitochondrial DNA. The accumulated mitochondrial DNA provokes inflammation through TLR9-dependent signaling and leads to heart failure. Drp1-mediated mitophagy is critical for the protection of the heart during pressure overload. TAT-Beclin 1 can activate both general and alternative mitophagy. Parkin has also been shown to enhance autophagy during pressure overload. The figure was created with Biorender.com

When mitophagy takes place, mitochondrial DNA is degraded by DNase II in lysosomes. DNase II hydrolyzes both exogenous DNA and mitochondrial DNA at low pH. Accumulation of mitochondrial DNA due to downregulation of DNase II can accelerate heart failure in the presence of pressure overload by activating TLR9-dependent inflammation [131]. TLR9 receptor-deficient mice have better cardiac function than wild-type mice under pressure overload conditions [131]. Whether interventions that upregulate mitophagy during pressure overload coordinately regulate degradation of mitochondrial DNA by DNase II or whether a mismatch occurs remains to be clarified. The effect of mitophagy during hypertrophy and heart failure is schematically represented in Fig. 4.

#### Mitophagy in diabetic cardiomyopathy

Obesity and diabetes are prevalent metabolic disorders that commonly induce diastolic dysfunction, hypertrophy, and inflammation in the heart, a condition collectively called diabetic cardiomyopathy [173]. Obesity and diabetes also contribute to the pathogenesis of heart failure with preserved ejection fraction (HFpEF), one of the most common forms of heart failure in developed countries [173]. Diabetic cardiomyopathy is associated with a shift in substrate utilization from glucose to fatty acids due to insulin deficiency. Increased utilization of fatty acids for energy production induces more oxidative stress and ultimately causes mitochondrial damage [74]. Mitochondrial dysfunction also causes an imbalance between fatty acid uptake and catabolism, leading to accumulation of lipid droplets and lipotoxicity [26]. Increasing lines of evidence suggest that mitophagy plays a critical role in the maintenance of mitochondrial function in diabetic hearts [227].

In mouse models of type 1 diabetes mellitus (T1DM), including OVE26 mice and streptozotocin (STZ)-treated mice, general autophagy is inhibited in the heart [37, 201, 204]. High glucose levels directly inhibit autophagy in cardiomyocytes [76]. Despite downregulation of general autophagy, however, mitophagy is stimulated in the T1DM mouse heart [204], accompanied by low levels of PINK1 and Parkin and high levels of the small GTPase Rab9, suggesting that alternative mitophagy is activated in these hearts [204]. Activation of mitophagy in the T1DM mouse heart plays an important role in maintaining mitochondrial function. Whether inactivation of general autophagy and activation of mitophagy are coordinately regulated in T1DM hearts, and, if so, how are currently unknown.

In mouse models of type 2 diabetes (T2DM), autophagy is activated in the heart, but its activation is transient and general autophagy is often inhibited during the chronic phase of T2DM [181]. For example, in mice fed a HFD, activation of general autophagy in the heart peaks at 6 weeks and declines thereafter. Interestingly, however, mitophagy remains activated after general autophagy is inactivated [181]. Thus, in both T1DM and T2DM, although general autophagy is inactivated during the chronic phase, mitophagy is activated even after general autophagy is inhibited. How is general autophagy inactivated during the chronic phase of diabetic cardiomyopathy? One mechanism could be activation of a negative regulator of autophagy, such as Mst1. It is also possible that continuous activation of autophagy leads to depletion of the cellular materials required for autophagosome formation. What is the underlying mechanism of mitophagy in T1DM and T2DM hearts that occurs when general autophagy is downregulated? We and others have shown that mitophagy in the T2DM heart is mediated through Parkin-dependent conventional mechanisms during early stages of HFD consumption, when mechanisms commonly used by conventional autophagy are available [144, 181]. However, alternative mitophagy, utilizing Ulk1-Rab9 mechanisms, predominates after conventional autophagy is inactivated [181]. Importantly, loss of mitophagy function consistently exacerbates mitochondrial dysfunction and cardiomyopathy in mice fed a HFD, during both the acute and chronic phases [179–181]. Furthermore, we have shown that mitophagy can be stimulated with TAT-Beclin 1 regardless of the timing of HFD consumption and that the enhancement of mitophagy is protective in T2DM hearts [179–181]. These results suggest that mitophagy plays an essential role in maintaining mitochondrial function and cardiac function in T2DM hearts. Mitochondrial dysfunction is commonly observed in the T2DM heart despite activation of mitophagy, indicating that the endogenous level of mitophagy is insufficient to prevent the progression of mitochondrial dysfunction and cardiomyopathy in these hearts. By the same token, the fact that stimulation of mitophagy alleviates cardiomyopathy regardless of the timing of HFD consumption indicates that interventions enhancing the level of mitophagy are effective in alleviating the progression of diabetic cardiomyopathy.

**Fig. 5:**
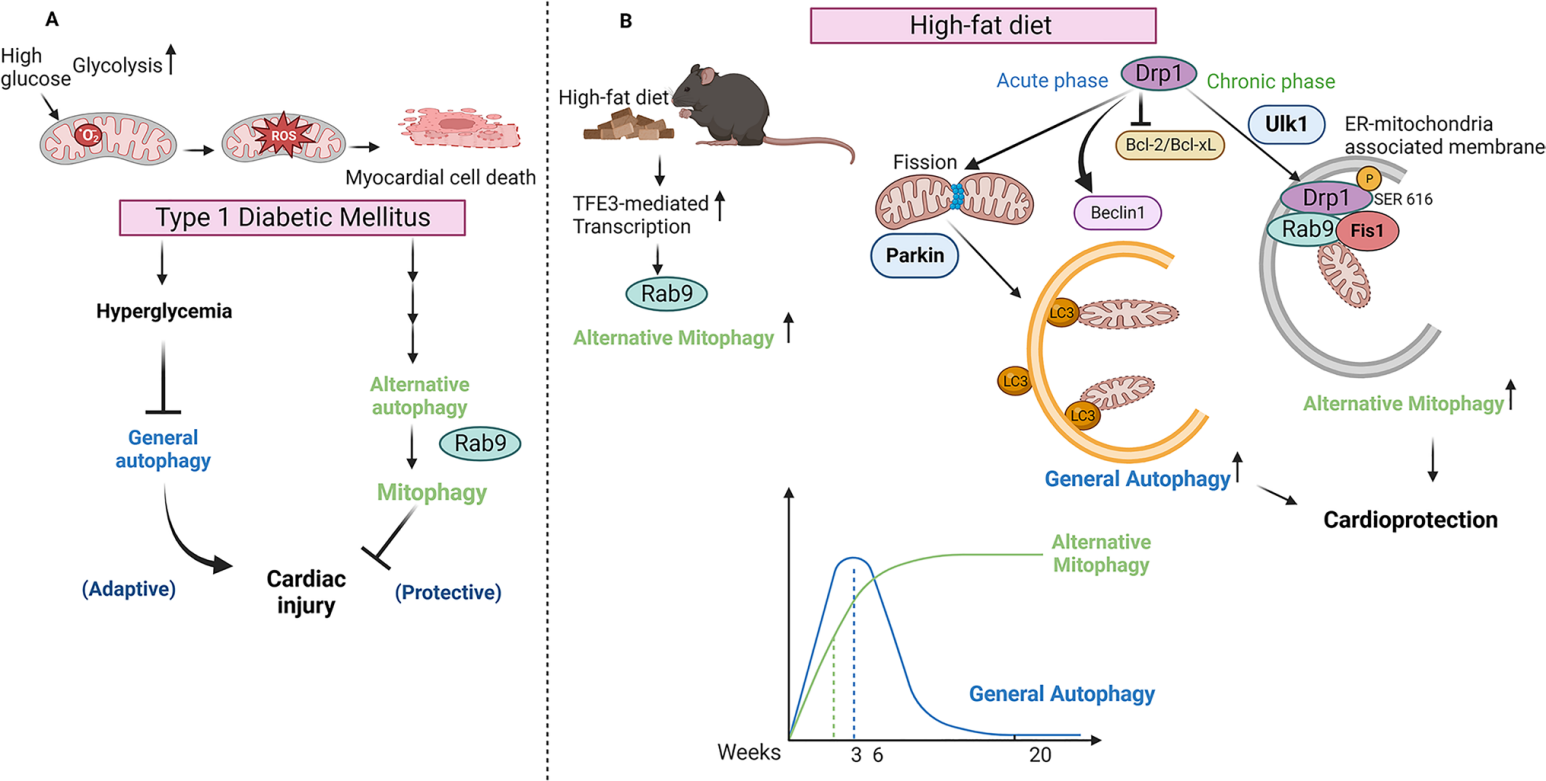
Mitophagy in diabetic cardiomyopathy. **A** Accumulated glucose elevates mitochondrial superoxide levels in type 1 diabetes and leads to myocardial cell death. While general autophagy is inhibited by high glucose levels, a compensatory mechanism simultaneously activates alternative autophagy and mitophagy and is cardioprotective. **B** In a mouse model of type 2 diabetes, mitophagy is stimulated in the heart through multiple mechanisms in a time-dependent manner. During the acute phase of HFD consumption, mitophagy is activated by Atg7- and Parkin-dependent mechanisms. Drp1 is also involved in mitophagy during the acute phase of HFD consumption by inhibiting Bcl-2/Bcl-xL interaction with Beclin 1, which allows activation of Beclin 1. In the chronic phase of HFD consumption, Drp1 is phosphorylated at Ser616 through unknown mechanisms and localized to ER–mitochondria-associated membrane, where it activates Rab9-mediated alternative mitophagy. These mechanisms may be compensatory for the downregulation of conventional mitophagy. Despite activation of conventional mitophagy in the acute phase and alternative mitophagy during the chronic phase, the level of mitophagy appears to be insufficient, and thus mitochondrial dysfunction in the heart develops during the chronic phase of type 2 diabetes. The figure was created with Biorender.com

Since T2DM is a chronic disease, it is possible that conventional autophagy has already been downregulated by the time diabetic cardiomyopathy develops in these patients. Thus, it is important to understand the molecular mechanism through which alternative mitophagy is activated during the chronic phase of T2DM. We found that transcription factor binding to IGHM enhancer 3 (TFE3), a member of the transcription factor EB (TFEB) family, is upregulated in the heart following 12 weeks of HFD consumption, concurrent with the time of observation of alternative mitophagy [180]. TFE3 is upregulated in the nuclear fraction of cardiomyocytes in response to HFD consumption and associates with the promoter region of the Rab9 gene [180]. Alternative mitophagy is inhibited during the chronic phase of HFD consumption in mice with cardiac-specific knockout of TFE3, indicating that alternative mitophagy requires TFE3 and transcription of Rab9 [180]. Thus, a better understanding of TFE3 may allow development of novel interventions to improve mitochondrial quality control mechanisms during the chronic phase of T2DM. We also found that Drp1 is phosphorylated at Ser616 during the chronic phase of HFD consumption and participates in stimulation of alternative mitophagy at the MAM [179]. Phosphorylation of Drp1 at Ser616 was also observed in human patients with obesity (Body mass index > 30 kg/m^2^) [179]. Thus, Drp1 could be another promising target to modulate mitophagy during the chronic phase in the T2DM heart. The effects of mitophagy during diabetic cardiomyopathy is schematically represented in Fig. 5.

### Perspectives and conclusions

The mechanisms of mitophagy are complex and require tightly regulated molecular signals. The activation mechanisms appear to differ between various cellular stresses and utilize multiple systems to tag and sequester the damaged mitochondria for degradation in the lysosome. Moreover, mitochondrial degradation can also occur through several mechanisms besides the authentic mechanism of mitophagy, including the endosomal–lysosome pathway, mitochondrial-derived vesicles, and autophagic secretion [178]. Therefore, in-depth studies using multiple approaches are required to understand both the mechanisms and the functional consequences of mitophagy in the heart. We believe that it is of high priority to address the following issues:

1. Are the mechanisms of mitophagy in cardiomyocytes identical to those in other cell types? Owing to the unique characteristics of cardiomyocyte mitochondria, including the presence of interfibrillar mitochondria, which affect mitochondrial dynamics [140], mitophagy in cardiomyocytes may not be identical to that in other cell types.
2. What is the specific role of ubiquitin-dependent and -independent (receptor-mediated) mitophagy in cardiomyocytes under various physiological and disease/stress conditions? How does the heart coordinate the use of the multiple mechanisms of mitophagy? What is the molecular mechanism mediating non-canonical mechanisms of mitochondrial degradation, including alternative mitophagy, endosomal mechanisms, microautophagy, and autophagic secretion of mitochondria? Although these mechanisms are often compensatory, they can be a major driver of mitochondrial quality control during chronic disease conditions, where conventional mechanisms of mitophagy are no longer available.
3. Stress-induced activation of the conventional mechanisms of mitophagy, including the PINK1–Parkin mechanism, is often transient. What is the mechanism that induces rapid inactivation of mitophagy in the heart? Investigating the role of signaling molecules negatively affecting autophagy (Mst1, mTOR, deubiquitinases and TNIP), transcriptional regulators of autophagy (CLOCK, KLF4, ATF4 and TFEB), and other mechanisms promoting rapid exhaustion of the autophagy machinery, such as LC3, is of great interest.
4. What is the consequence of insufficient mitophagy? What are the roles of the mitochondrial DNA sensing mechanism, including ZBAP1 and cGAS [89], and the innate immune responses/inflammation?
5. Can excessive mitophagy take place? What is the consequence of excessive mitophagy? Unchecked mitochondrial fission and mitophagy exacerbate doxorubicin-induced cardiomyopathy [13]. Whether the detrimental effect of mitophagy takes place under other cardiac stress conditions needs to be clarified.
6. What is the role of mitophagy in other cell types during heart failure? Suppression of mitophagy in cardiac fibroblasts alleviates cardiac fibrosis [225]. Thus, mitophagy may not be equally salutary in every cell population in the failing heart.
7. What is the extent and the functional significance of mitophagy in human hearts during cardiac stress?

To address these issues, it is important to establish a more convenient assay system to accurately evaluate the level of mitophagy in the heart and the various cell types therein under stress conditions. Elucidating the underlying signaling mechanism affecting the level of mitophagy should provide important clues to improve mitophagy and mitochondrial quality control mechanisms under given conditions. Eventually, development of small molecule regulators of mitophagy would have a remarkable impact, since insufficient mitophagy appears to be a common trigger of heart failure in almost every cardiovascular condition.
